# Vagus nerve stimulation (VNS): recent advances and future directions

**DOI:** 10.1007/s10286-024-01065-w

**Published:** 2024-10-04

**Authors:** Christopher W. Austelle, Stewart S. Cox, Kristin E. Wills, Bashar W. Badran

**Affiliations:** 1 Department of Psychiatry and Behavioral Sciences, Stanford University, 401 Quarry Road, Palo Alto, CA 94305 USA; 2 Veterans Affairs Palo Alto Healthcare System, and the Sierra Pacific Mental Illness, Research, Education, and Clinical Center (MIRECC), Palo Alto, CA USA; 3 Department of Psychiatry and Behavioral Sciences, Medical University of South Carolina, Charleston, SC USA

**Keywords:** Vagus nerve stimulation, VNS, Vagus nerve, Brain stimulation, Neuromodulation

## Abstract

**Purpose:**

Vagus nerve stimulation (VNS) is emerging as a unique and potent intervention, particularly within neurology and psychiatry. The clinical value of VNS continues to grow, while the development of noninvasive options promises to change a landscape that is already quickly evolving. In this review, we highlight recent progress in the field and offer readers a glimpse of the future for this bright and promising modality.

**Methods:**

We compiled a narrative review of VNS literature using PubMed and organized the discussion by disease states with approved indications (epilepsy, depression, obesity, post-stroke motor rehabilitation, headache), followed by a section highlighting novel, exploratory areas of VNS research. In each section, we summarized the current role, recent advancements, and future directions of VNS in the treatment of each disease.

**Results:**

The field continues to gain appreciation for the clinical potential of this modality. VNS was initially developed for treatment-resistant epilepsy, with the first depression studies following shortly thereafter. Overall, VNS has gained approval or clearance in the treatment of medication-refractory epilepsy, treatment-resistant depression, obesity, migraine/cluster headache, and post-stroke motor rehabilitation.

**Conclusion:**

Noninvasive VNS represents an opportunity to bridge the translational gap between preclinical and clinical paradigms and may offer the same therapeutic potential as invasive VNS. Further investigation into how VNS parameters modulate behavior and biology, as well as how to translate noninvasive options into the clinical arena, are crucial next steps for researchers and clinicians studying VNS.

## Introduction

Vagus nerve stimulation (VNS) is an exciting modality that has already demonstrated potential to treat a variety of medical and neuropsychiatric disorders. Historically, VNS was found to be an effective treatment for epilepsy, and later, developed as a treatment for major depression [1]. Over the past several decades, VNS has gained approvals in other areas, including obesity, post-stroke motor rehabilitation, and migraine [1]. With increasing interest in VNS for myriad conditions, ongoing investigations will likely lead to additional approvals. This review serves to give readers an overview of the science leading to the current clinical indications of VNS, along with the current state of research that may guide the field to future approved indications.

VNS is in prime position to gain additional approvals for neuropsychiatric disorders over the coming years. As outlined in the following sections, we will discuss the current state of VNS for approved indications as well as highlight future areas for growth. There is a plethora of evidence supporting the potential for VNS to treat a variety of disorders, including neurological and psychiatric disorders [2–4], inflammatory and immune disorders [5, 6], pain-related disorders [7], cardiovascular diseases [8], and other diseases related to autonomic dysfunction [9].

The vagus nerve has many functions and innervates various end-organs throughout the chest and abdomen, which highlights its therapeutic potential for a variety of conditions [1]. But access to VNS has been limited over the first few decades of use due to the invasive and expensive nature of implanted VNS. The recent development of noninvasive forms of VNS have opened the door for scientists to study the effects of vagal stimulation more closely [10, 11]. Additionally, closed-loop VNS systems are beginning to make their way into research and clinical domains [12, 13]. In this era of personalized medicine, researchers are studying ways to make VNS treatments individualized and unique. There is still much for us to learn about this modality. In the following sections, we will further highlight and explore the current gaps in the literature, in addition to the ones already described here.

## Early history of VNS

**Fig. 1:**
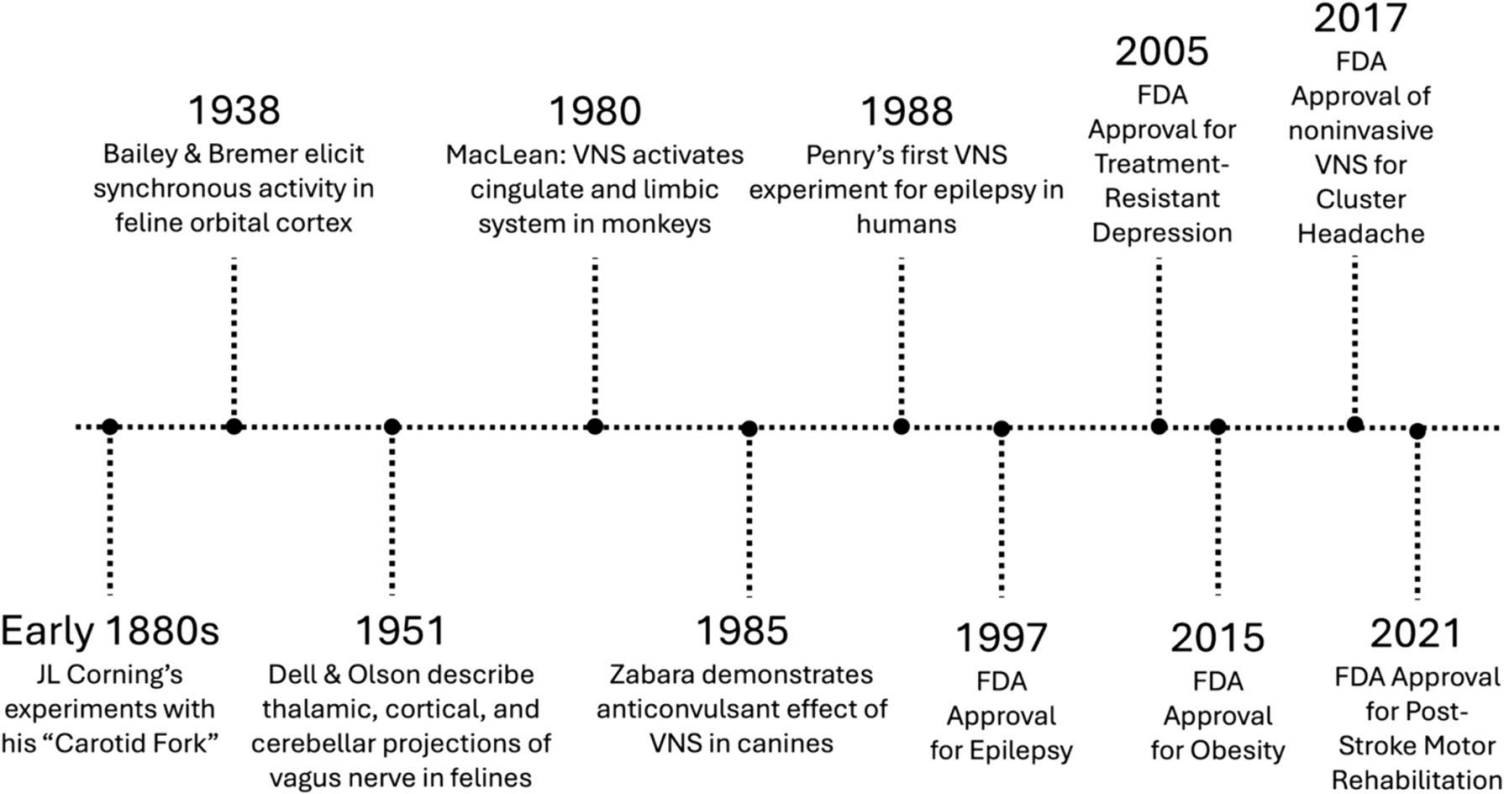
History of VNS timeline showing the early VNS experiments in animal models, leading to the first experiment in humans. Over the past few decades, VNS has gained FDA approval for multiple diseases, including medication-refractory epilepsy, treatment-resistant depression, obesity, cluster headache, and post-stroke motor rehabilitation. As noted in the figure, all of the FDA approvals are for implanted VNS with the exception of the approval for cluster headache (which is for a form of noninvasive VNS called transcutaneous cervical vagus nerve stimulation, or tcVNS)

The earliest attempt to administer electricity to the vagus nerve came from New York in the late 1800s (see Fig. 1). A neurologist named James Leonard Corning invented a fork-like device to treat epilepsy based on the contemporary idea that seizures were due to abnormal cerebral blood flow [14]. The “Corning fork” functioned via two mechanisms: (1) mechanical compression and (2) electrical stimulation of the carotid sheath. Although the method fell out of favor, interest in electrical stimulation of the vagus nerve was revived in the twentieth century. A series of trials in the early twentieth century began to elucidate the neural effects of electrically stimulating the vagus nerve [15, 16]. Following Bailey and Bremer’s seminal trial that demonstrated the ability of VNS to synchronize activity in the feline cortex [15], Dell and Olson demonstrated the localized effects of VNS in the feline brain [16]. Building on prior experiments that started decades earlier, MacLean demonstrated the ability of VNS to modulate activity in the cingulate cortex in primates in 1980 [17].

The modern invasive VNS device, developed by Zabara and colleagues in the 1980s, has three main components: (1) a pulse generator that is implanted subcutaneously in the patient’s chest wall, (2) an electrode cuff wrapped around the cervical bundle of the left vagus nerve, and (3) a wire connecting the pulse generator and electrode cuff [18]. A surgeon performs the implantation in an ambulatory setting and, after 2 weeks, activates the device. Over the following weeks and months, a specialized provider (usually a neurologist or psychiatrist) programs the device, adjusting parameters to treat the patient’s symptoms and minimize side effects [19].

Since the 1980s, the science supporting the therapeutic potential of implanted VNS (iVNS) has been steadily growing for a variety of medical and neuropsychiatric illnesses, including epilepsy, depression, obesity, headaches, other pain-related disorders, inflammatory disorders, and cardiovascular disease. Most of our current understanding comes from animal models, while further study of these indications in humans has been limited by the expensive and invasive nature of the implanted cervical device. In recent years, novel noninvasive VNS methods have been developed, offering the opportunity to translate work from animal models to humans [23]. To date, two forms of noninvasive VNS have being used for clinical and research purposes. One of the devices involves the noninvasive stimulation of the vagus nerve through the neck, a technique known as transcutaneous cervical vagus nerve stimulation (tcVNS). The other device stimulates the auricular branch of the vagus nerve (ABVN) via electrodes that are placed on the ear, known as transcutaneous auricular vagus nerve stimulation (taVNS). Although believed to mimic the effects of iVNS, it is still unclear whether iVNS and taVNS or tcVNS produce identical effects. When applicable, this review will specify which of the forms of VNS (iVNS, taVNS, or tcVNS) are used in the sections below.

## Mechanisms of VNS

The vagus nerve is the tenth cranial nerve and travels from the brainstem, through the neck, and innervates organs throughout the chest and abdomen [20]. The nerve is present bilaterally and is an essential component of the parasympathetic nervous system. Around 80% of the nerve’s fibers are afferent, carrying sensory information from the viscera to the brain. The various functions of the vagus can be targeted using electrical stimulation, eliciting central and peripheral effects [20]. Due to its widespread projections and various functions, the mechanism of therapeutic action of VNS for any given disorder is likely multifactorial, with multiple mechanisms working synergistically. For example, major depression is associated with alterations in the brain and periphery, including immune and endocrine dysfunction [21]. VNS has been shown to modulate neural activity as well as modulate the neuroendocrine–immune axis and function [5]. Thus, it is likely that there are multiple mechanisms by which VNS exerts its effects in depression alone. This section will give readers an overview of all the potential mechanisms of VNS. Implanted VNS and noninvasive VNS have similar mechanisms, although the difference between the two modalities is not well understood as there has never been a head-to-head trial comparing their effects.

VNS produces anticonvulsant effects, first discovered by Zabara in 1985 [18]. While the antiepileptic mechanisms of VNS are not fully understood, evidence suggests that norepinephrine plays a crucial role [22]. The vagus nerve projects through the locus coeruleus, or the major noradrenergic nucleus in the brain. In a rodent model, researchers ablated the locus coeruleus and found that VNS lost its anti-seizure effects [22]. Other mechanisms likely contribute to the anticonvulsant effects of VNS, but more data are needed.

As highlighted in the previous paragraph, VNS modulates the monoamine system (e.g., serotonin, norepinephrine). VNS has been shown to increase the concentration of monoamines found in the CSF [23]. In rodent models, VNS increases the firing rate of norepinephrine and serotonin neurons [24]. This mechanism likely plays a key role in the antidepressant effect of VNS. Additionally, neuroimaging studies have demonstrated that VNS modulates key areas of the brain involved in regulating mood, including the prefrontal cortex and limbic system [5].

Animal models have also been helpful in elucidating the neural plasticity-enhancing effects of VNS. VNS increases the expression of brain-derived neurotrophic factor (BDNF) and synaptic spine density in the hippocampus [25]. This mechanism likely plays a role in the mechanism of VNS for multiple disorders, including depression and post-stroke motor rehabilitation [26]. When paired with a motor task, VNS reorganizes the primary motor cortex and increases the representation of behavioral tasks in associated brain regions [27]. The same concept has been demonstrated in non-motor paradigms, where VNS paired with an auditory tone reorganizes and increases plasticity within the primary auditory cortex [28].

VNS has also been shown to modulate immune function [29]. It decreases immune markers and inflammatory cytokines [5]. This has been shown to be correlated with the therapeutic effects of VNS for post-traumatic stress disorder (PTSD) [30]. VNS also exerts cardiovascular effects, closely related to modulation of the autonomic system. Acute trains of VNS decrease heart rate and other sympathetic markers [11]. VNS has also been shown to decrease the heart rate during the stress response [31]. Additionally, it has been shown to reduce sympathetic activity and improve baroreflex control [32, 33], which are likely mechanisms in treating conditions that are characterized by sympathetic overactivity. The gastrointestinal effects of VNS likely work through a concert of multiple systems along the brain–gut axis. Specifically, the gastrointestinal effects of VNS work closely in concert with the cholinergic anti-inflammatory pathway [34].

## VNS for epilepsy

### Implanted VNS

Interest in vagal stimulation re-emerged in the mid-twentieth century when researchers began investigating the cortical effects of iVNS in animal models. Researchers demonstrated that iVNS could synchronize and desynchronize cortical activity, and that these effects last beyond the duration of stimulation [5]. In the 1980s, Zabara and colleagues showed that vagal stimulation could abort chemically induced seizures and tremors in canines [18]. Later in that decade, the same group developed the first modern iVNS device, the NeuroCybernetic Prosthesis System, which was first implanted in human subjects in 1987 [19]. Early pilot trials assessing the impact on epilepsy showed that iVNS was effective in reducing refractory partial seizure frequency in patients who had not previously responded to medications (see Table 1) [19].

**Table 1:** VNS for epilepsy trials

Authors	Study/sample size	Parameters (active group)	Implanted/ transcutaneous	Primary outcome		Study conclusions
Penry and Dean [19]	4 patients with seizure disorder	PW: 47–50 Hz	Implanted		Long-term safety and efficacy	All patients tolerated the procedure with minimal long-term side effects. Seizures were reduced in 3/4 of patients
		On/off: varied				
		I: 1–3 mA				
Uthman et al. [123]	14 patients with medically refractory partial seizures	PW: 250 μs	Implanted		Mean reduction in seizure frequency	Mean reduction in seizure frequency was 46.6% after 14–35 months; 14 of 35 patients showed at least 50% reduction in seizure frequency
		Freq: 50 Hz				
		On/off: 60 s/60 min				
		I: 1 mA				
Ben-Menachem et al. [35]	Multicenter prospective randomized, parallel, double-blind study of 67 patients with refractory partial seizures	PW: 300 μs	Implanted		Comparison of high- vs. low-stimulation VNS on seizure frequency	Significantly more patients experienced reduced seizure frequency in the group receiving therapeutic VNS than in those receiving low/non-therapeutic treatment. Mean seizure frequency was significantly reduced with therapeutic VNS
		Freq: 30 Hz				
		On/off: 30 s/5 min				
		I: 1.5 mA				
Handforth et al. [36]	Multicenter, add-on, double-blind, randomized, active-control study of 196 patients with partial or generalized secondary seizures	PW: 500 μs	Implanted		Comparison of high- vs. low-stimulation VNS on seizure frequency	Patients receiving high-stimulation VNS showed significantly greater seizure reduction than low-stimulation group
		Freq: 30 Hz				
		On/off: 30 s/5 min				
		I: < 3.5 mA				
Amar et al. [37]	18 patients with medically refractory epilepsy and at least six complex partial or secondarily generalized seizures per month	High-level group:	Implanted		Comparison of high-stimulation VNS vs. low-stimulation on seizure frequency	High-stimulation VNS patients had significantly greater reduction in seizure frequency at 3- and 18-month follow-up
		PW: 500 μs				
		Freq: 30 Hz				
		On/off: 30 s/5 min				
		I: up to 3.5 mA				
		Low-level group:				
		PW: 130 μs				
		Freq: 1 Hz				
		On/off: 30 s/3 h				
		I: up to 3.5 mA				
Scherrmann et al. [38]	95 adult patients with drug-resistant seizures who had received implants	PW: 500 μs	Implanted		Median percentage of reduction in seizure frequency compared to antiepileptic drug alone	Median percentage of reduction in seizure frequency as compared to baseline was 30%, and seizure outcome was positively correlated with VNS duration
		Freq: 20 Hz				
		On/off: 7 s/30 s				
		I: 0.25–3 mA				
DeGiorgio et al. [39]	Multicenter, randomized trial of three unique modes of VNS, which varied primarily by duty cycle; 64 total subjects with at least one seizure/30 days with alteration of consciousness	PW: 250–500 s	Implanted		Within-group and between-group percentage changes in seizure frequency in order to assess optimal device settings	Patients in all three groups experienced a significant reduction in cumulative seizure frequency during the 3-month treatment period; no statistical difference was seen between groups
		Freq: 20–30 Hz				
		On/off: 7–30 s/18 s–3 min				
		I: 0.87–0.93 mA				
Klinkenberg et al. [40]	41 children with either partial or generalized epilepsy	PW: 0.5 ms	Implanted		Comparison of high-stimulation VNS vs. low-stimulation on seizure frequency and severity in children	VNS well tolerated in children, but no significant difference seen in frequency or severity between low and high stimulation groups
		Freq: 30 Hz				
		On/off: 30 s/5 min				
		I: 0.25 mA				
Dibué et al. [42]	Meta-analysis of a total of 480 patients with Lennox–Gastaut syndrome	Varied by study	Implanted		Proportion of responders (at least 50% reduction in seizure frequency)	54% of patients responded to adjunctive VNS therapy, and treatment was well tolerated
Tzadok et al. [44]	46 patients aged 5–31 years with epilepsy	Individually dosed	Closed-loop implanted		VNS stimulation deliveries in response to increased heart rate (predictor of immanent seizure)	Patients defined as responders (at least 50% reduction in seizure frequency) was 60.9%, 10.9% experiencing complete seizure freedom at 13-month follow-up
Stefan et al. [45]	10 patients with pharmacoresistant epilepsy	PW: 300 μs	Transcutaneous		Assessment of t-VNS safety and tolerability as alternative treatment option in pharmacoresistant epilepsy	Of the seven patients who completed the study, an overall reduction in seizure frequency was observed in five patients after 9 months and was found to be safe and well tolerated
		Freq: 10 Hz				
He et al. [46]	14 pediatric patients with intractable epilepsy	Freq: 20 Hz	Transcutaneous		Baseline seizure frequency was compared vs. 8 weeks, weeks 9–16. and weeks 17–24, using seizure diaries	The mean reduction in seizure frequency relative to baseline was 31.83% after week 8, 54.13% from weeks 9 to 16, and 54.21% from week 17 to the end of week 24
		I: 0.4–1 mA				
Aihua et al. [47]	60 patients with pharmacoresistant epilepsy	PW: 0.2 s	Transcutaneous		Seizure frequency according to the patient’s seizure diary after 12 months in active vs. sham group	Monthly seizure frequency was significantly lower in the treatment group than in the control group
		Freq: 20 Hz				
		On/off: continuous 20 min				
		I: Directed by patient				
Rong et al. [49]	RCT of 144 patients with refractory epilepsy	PW: 1 ms	Transcutaneous		Reduction in seizure frequency using the modified Engel scale in active vs. sham group	After 8 weeks, the active group had significantly greater seizure reduction than the sham group
		Freq: 20–30 Hz				
		I: 1 mA				
Bauer et al. [50]	RCT of 76 patients with resistant epilepsy	PW: 250 μs	Transcutaneous		Demonstrated superiority of add-on therapy with tVNS vs. active control in reducing seizure frequency over 20 weeks	Significant reduction in seizure frequency was found in patients in the active tVNS group compared to the active controls
		Freq: 25 Hz				
		On/Off: 30 s/30 s				
Liu et al. [51]	17 patients with refractory epilepsy	PW: 200 s	Transcutaneous		Investigation of efficacy of tVNS via assessment of the frequency of seizures at 3 and 6 months	The frequency of seizures decreased in 13/17 subjects, with an average rate reduction of 31.3% at 3 months. Following 6 months, the frequency decreased in 16/17 subjects, with an average rate reduction of 64.4%
		Freq: 10 Hz				
		Time: 20 min 3×/day				
		I: 4 mA, 2 mA increase weekly				

*PW* pulse width

Epilepsy is one of the most common neurological illnesses in the world, affecting around 50 million people globally [14]. Five million people are newly diagnosed with epilepsy every year. The incidence of refractory epilepsy remains high; 20–40% of newly diagnosed epilepsy cases become refractory to treatment [14]. Additionally, there is a high rate of psychiatric comorbidity in this patient population. The development of novel and accessible interventions for this group of disorders is clearly needed.

Several multicenter, double-blind randomized controlled trials (RCTs) were critical in iVNS gaining Food and Drug Administration (FDA) approval. In the first double-blind RCT investigating iVNS for partial epilepsy [35], a cohort of 114 patients were randomized into high- and low-frequency stimulation arms and followed for a period of 3 months (Table 1). At the end of the study, a total of 31% of all the participants in this study were responders, defined as experiencing greater than 50% reduction in seizures. Specifically, the high-frequency group experienced a 25% reduction in seizures, while the low-frequency group’s seizures decreased by 6%.

Subsequently, Handforth and colleagues performed a similar experiment, investigating high- versus low-frequency stimulation for the treatment of partial epilepsy [36]. The results from this trial confirmed previous findings, showing a significant difference in reduction of seizures between the high-frequency (28% reduction in seizures) and low-frequency (15% reduction) groups. A smaller RCT (*N* = 17) from Amar and colleagues similarly demonstrated the efficacy of iVNS in reducing seizures in patients suffering from intractable epilepsy, with a response rate of 57% [37]. Additionally, a pair of non-blinded RCTs on iVNS had significant success in reducing seizures in patients who had not responded to antiepileptic medications [38, 39].

Schermann and colleagues found similar reduction rates (about 30%) as the RCTs, with a 45% response rate [38]. They also found that a standard duty cycle (stimulation-on period of 30 s) had better outcomes than a rapid cycle (stimulation-on period of 7 s). The effect of duty cycle on outcomes in epilepsy patients is still unclear. However, DeGiorgio found that a duty cycle of 30 s on, 3 min off for the first 3 months of iVNS treatment produced the most beneficial outcomes [39]. They also confirmed that a higher duty cycle and shorter off time may be required for patients who are refractory to iVNS.

Based on its efficacy for partial seizures in adults, additional studies later worked to expand upon these findings in other populations. For example, Klinkenberg and colleagues ran the first double-blinded RCT investigating the effectiveness of iVNS in treating pediatric seizures [40]. A group of 35 pediatric participants with intractable partial or generalized seizures were randomized to receive 20 weeks of high- or low-output stimulation. In the high-output group, current amplitude could be titrated to a maximum of 1.75 mA, while the low-output group was limited to 0.25 mA. After the initial double-blinded 20-week period, all participants then received high-output stimulation for an additional 19 weeks [40]. There was no significant difference between the high- and low-output groups at the end of the double-blind period; after the additional 19 weeks of high-output treatment, however, 26% of all participants experienced a reduction in seizure frequency by 50% or more.

In all the trials discussed, patients continued to take antiepileptic medications as prescribed, with dosing and blood levels maintained at appropriate therapeutic doses throughout. There is no evidence suggesting that iVNS interferes or interacts with medications commonly prescribed for epilepsy. Based on the positive findings in the aforementioned trials, iVNS was approved by the FDA in 1997 for the treatment of partial-onset seizures that are refractory to medications [41]. Further success in studies assessing the reduction of seizure burden in younger patients has since allowed approval for reducing seizures to include younger populations as young as 4 years of age. As iVNS continues to be explored as an effective modality in epilepsy, one can remain hopeful that approval for iVNS may expand to other types of seizures. The data from the four RCTs discussed earlier in this section are mainly related to partial seizures; however, there is growing evidence that iVNS may be effective in reducing the frequency of other types, evidenced by the positive outcomes seen in populations who experience a range of seizure types like those with Lennox–Gastaut syndrome [42].

There are several intriguing new developments related to iVNS treatment of epilepsy. One such advancement is closed-loop iVNS systems, or devices which deliver stimulation in response to a predefined input (e.g., a change in parameters from normal physiological functioning). Recent applications of closed-loop iVNS systems for epilepsy have focused on delivering stimulation in response to ictal tachycardia, or an increase in heart rate from baseline that is associated with seizures [43]. As more than 75% of patients with epilepsy experience ictal tachycardia [43], closed-loop iVNS offers a promising solution to a common deleterious outcome by stimulating the vagus nerve when tachycardia events are detected. These systems (see [44] for more details) have already begun to be implemented in clinical settings.

### Noninvasive VNS

Another recent advancement stems from the emergence of noninvasive VNS devices. Transcutaneous cervical VNS (tcVNS) and transcutaneous auricular transcutaneous VNS (taVNS) are beginning to be studied as potential antiepileptic treatments. The data remain limited, as these devices are still in their infancy, making it difficult to predict whether these modalities will be effective for epilepsy in the same way as iVNS. After early pilot trials demonstrated safety, feasibility, and preliminary efficacy [45, 46], the first controlled trials of transcutaneous VNS for pharmacoresistant epilepsy were undertaken (see Table 1). Aihua and colleagues recruited children and adults with medication-refractory epilepsy for a 12-month randomized controlled trial using taVNS [47]. After 1 year, the monthly seizure frequency was significantly lower in the active group than in the control group. Additionally, all 60 patients who were enrolled in the study showed improvements in anxiety, depression, and quality of life self-reports. Side effects were minimal and self-limited.

Rong and colleagues completed a multicenter taVNS RCT in 2014, which recruited 50 patients older than 12 years with drug-resistant epilepsy [48]. Of the 50 enrolled, 47 patients finished the study. After 8 weeks of treatment, 12% of participants were seizure-free and 24% had a reduction in seizure frequency. After 24 weeks, the number of patients who were seizure-free rose to 16%, while 38% had reduced seizure frequency. In another multicenter RCT led by Rong, 144 patients with pharmacoresistant epilepsy were randomized to 8 weeks of blinded and controlled taVNS [49]. Participants received another 16 weeks of active stimulation for a total of 24 weeks. At 8 weeks, 41% of patients in the active taVNS group had experienced a reduction in seizures (compared to 27.5% in the control group). After 24 weeks (including 16 weeks of active stimulation for both groups), 47.7% of the active group and 47.5% of the initially control group saw reductions in seizures.

In 2016, Bauer and colleagues reported on the only double-blinded multicenter RCT to date [50]. In this trial, 76 patients with resistant epilepsy were randomized to receive either 25 Hz or 1 Hz taVNS. After 20 weeks, responder rates were similar in both groups (25%, 50%), and adherence to the treatments was high (84% in the 1 Hz group and 88% in the 25 Hz group). There was a significant reduction in seizure frequency in the 25 Hz group as well. Other trials have confirmed the findings of these promising early trials, including Barbella and Liu [51, 52]. More recently, Yang reported on an RCT that recruited 150 patients with drug-resistant epilepsy to receive 20 weeks of double-blind active or sham taVNS. After 20 weeks, the active group’s relative reduction in seizure frequency was higher than that in the control group. Secondary measures of anxiety, depression, cognition, and quality of life did not reveal any significant differences between the active and control groups. In all the trials discussed, transcutaneous VNS was found to be tolerable and to generate minimal side effects.

## VNS for depression

### Implanted VNS

Major depression is a common diagnosis and a leading cause of disability in the United States and worldwide. Over the past two decades, rates of depression have risen, and annual prevalence of a major depressive episode is up to 7.1% [32]. A significant portion of patients (around 30%) who suffer from depression do not respond to current first-line treatments like psychotherapy and medications [53]. The burdens on the individual and society due to major depressive disorder (MDD) and treatment-resistant depression (TRD) are very costly; novel treatments are needed to tackle this growing problem. Psychiatrists have been interested in the potential antidepressant effects of iVNS due to several converging lines of evidence, including functional neuroimaging showing that iVNS modulates mood-regulating brain regions, success in the treatment of depression with other antiepileptic medications, and evidence that iVNS modulates the monoamine system [20]. Early open-label iVNS pilot trials in depressed patients were promising and were further supported by results from investigations into the mood effects of iVNS in patients with epilepsy [54, 55, 56].

Rush and colleagues led the first open-label pilot trial of iVNS for treatment of TRD (Table 2) [57]. The multisite study recruited 40 depressed participants with a history of MDD or bipolar disorder and who had failed to respond to multiple medication trials. Stimulation was turned on 2 weeks after implantation and titrated to the maximum tolerable level over the next 2 weeks. After 4 weeks post-implantation, parameters were left unchanged for the remaining 8 weeks. At 3 months, 40% of participants had at least a 50% reduction in depressive symptoms, per questionnaire (labeled “responders”). In an extension beyond the initial 3-month period, the study team recruited an additional 30 participants [54]. After 1 year of treatment with iVNS, the improvements seen after 3 months were sustained (40–46% response rate) and remission rates were significantly increased (29%) compared to controls. In a 2-year naturalistic follow-up, Nahas and colleagues uncovered a common theme that was developing—iVNS produces a slow but sustained antidepressant response (as measured by the Montgomery–Åsberg Depression Rating Scale, or MADRS) [58].

**Table 2:** VNS for depression trials

Authors	Study/sample size	Parameters (active group)	Implanted/transcutaneous	Primary outcome	Conclusions
Rush et al. [57]	30 patients with pharmacoresistant depression currently in major depressive episode	PW: 500 μs Freq: 20-30 Hz On/off: 30 s/5 min I: 0.25 mA	Implanted	Response rates (at least 50% reduction) in baseline clinical depression scores	Response rates to VNS were 40–50%
Sackeim et al. [54]	Open pilot study of 60 patients with treatment-resistant depressive episodes	PW: 500 μs Freq: 20-30 Hz On/off: 30 s/5 min I: 0.25 mA	Implanted	Response rates (at least 50% reduction) in baseline clinical depression scores	Response rates to VNS ranged from 30% to 37% after 10 weeks of VNS depending on depression scale assessed
Nahas et al. [58]	Open pilot study of 59 participants in a treatment-resistant major depressive episode	Individually dosed	Implanted	Response rates (at least 50% reduction) from the baseline Hamilton Depression Rating Scale (HAM-D) after 2 years of adjunctive VNS	HAM-D response rates were 31% after 3 months, 44% after 1 year, and 42% after 2 years of adjunctive VNS
Rush et al. [59]	RCT of 235 outpatients in major depressive episode	PW: 500 μs Freq: 20-30 Hz On/off: 30 s/5 min I: 0.25 mA	Implanted	Response rates (at least 50% reduction) from the baseline Hamilton Depression Rating Scale in active vs. sham VNS groups	No significant difference was seen in Hamilton Rating scale after 10 weeks; however, a significant reduction in self-reported depressive symptoms was seen in active VNS group vs. sham
George et al. [4]	Nonrandomized, prospective comparison of 329 participants with treatment-resistant depression	PW: 500 μs Freq: 20-30 Hz On/off: 30 s/5 min I: 0.25 mA	Implanted	Change in improvement in Inventory of Depressive Symptomatology self-report (IDSSR) and Hamilton Rating Scale for depression over 12 months between groups	VNS + treatment as usual (TAU) had significantly greater improvements on IDSSR per month across 12 months, and greater improvement on Hamilton scale at 12 months
Hein et al. [63]	37 patients with major depression	Freq: 1.5 Hz On: 15 min × 1–2 daily I: 130uA	Transcutaneous	Change from baseline in Hamilton Depression Rating Scale and the Beck Depression Inventory in active vs. sham tVNS groups	Mean decrease in the Beck Depression Inventory for the active group was 12.6 points, compared to 4.4 points in the sham group. HAM-D score did not change significantly in the two groups
Rong et al. [64]	91 patients with depression received tVNS for 12 weeks. In a second cohort, 69 patients received 4 weeks of sham tVNS followed by 8 weeks of tVNS	PW: 0.2 ms Freq: 20 Hz On/off: continuous 30 min/day I: 4-6 mA	Transcutaneous	Change from baseline in Hamilton Depression Rating Scale at 0, 4, 8, and 12 weeks of tVNS	After 4 weeks, patients in the tVNS group showed greater improvement vs. sham as indicated by Hamilton score changes as well at week 4, and this change continued until week 12 during tVNS
Li et al. [66]	107 patients with MDD randomly assigned to receive tVNS or citalopram	PW: 0.2 ms Freq: 20 Hz On/off: continuous 30 min × 2/day I: varied	Transcutaneous	Comparison of Hamilton Depression Rating Scale measured every 2 weeks between tVNS and citalopram group across 12 weeks	The Hamilton scores were reduced in both treatment groups; however, there was no significant group-by-time interaction

*PW* pulse width

In the first and only randomized and sham-controlled trial of iVNS for depression completed to date, 235 depressed participants who had previously failed treatment with medications received either active or sham iVNS treatment for 10 weeks [59]. After 10 weeks, there was no significant difference in response rates between the active and sham groups (15.2% in the active group, 10% sham). In the 1-year-follow-up (iVNS was turned “on” in the control group after 10 weeks, and parameters could be titrated as needed in both groups), researchers found that response and remission rates had grown in both groups [60]. This study had several flaws that likely limited the findings, the first being the 10-week double-blinded window. There is overwhelming evidence that iVNS induces its antidepressant effects slowly, and 10 weeks was likely too brief to capture the response. The study was also limited by dosing—the current could not be titrated above 1.0 mA and was likely underdosed.

In 2005, George and colleagues investigated the antidepressant effects of iVNS compared to treatment as usual. The study recruited patients with TRD and consisted of two arms: (1) iVNS with treatment as usual and (2) treatment as usual alone [4]. They found that TRD patients receiving iVNS with treatment as usual had a significantly higher response rate than the group receiving treatment as usual alone (27% for the adjunctive iVNS group, 13% for the treatment-as-usual group). These results, together with the results from the open-label trial and RCT, led to the FDA approval of iVNS for TRD in 2007.

The evidence supporting implanted cervical iVNS for depression is certainly exciting and offers promise to the large number of patients suffering from depression who have not responded to medications. However, access to iVNS has been limited since 2007 due to a non-coverage determination made by the Centers for Medicare & Medicaid Services (CMS), in part because of the nonsignificant results from the only RCT published to date [59]. There is renewed hope that access to iVNS for depression will be expanded due to a study known as the RECOVER trial. This is an ongoing multicenter RCT that is planning to enroll a total of 6800 patients with TRD. There will be two major differences compared to the first RCT: (1) the double-blind period in this trial will be 52 weeks (compared to 10 weeks in the only other RCT), and (2) dosing–current can be titrated up to 3 mA (compared to 1 mA in the other RCT). If positive, this trial would likely provide the evidence needed for CMS and other insurance companies to cover iVNS for TRD.

Several common themes have developed in the literature on VNS for depression and VNS for epilepsy. Firstly, the response to VNS appears to grow slowly over time [38]. Patients who initially respond to VNS, measured as a reduction in seizures or a decreased score on a depression scale, tend to sustain those benefits over time. Secondly, VNS is associated with improved quality of life in populations suffering from epilepsy and depression (as measured by the Quality of Life in Epilepsy Inventory [QOLIE] and Quality of Life Enjoyment and Satisfaction Questionnaire–Short Form [Q-LES-Q-SF]) [61, 62]. Interestingly, VNS is associated with increased quality of life in both responders and nonresponders [62]. The side effect profiles of VNS are similar in the epilepsy and depression literature [54]. These side effects tend to decrease over time and can also be decreased or resolved by adjustment of stimulation parameters.

### Noninvasive VNS

In addition to the cervical VNS studies, there have been several pilot studies investigating the potential of transcutaneous modes of VNS to treat patients suffering from depression. Noninvasive VNS has several advantages over iVNS, including cost and accessibility. Noninvasive VNS can be easily self-administered by patients at home, with minimal monitoring required. Additionally, noninvasive options make it easier to study biological and behavioral effects of vagal stimulation in humans and promise to streamline the translational pipeline from preclinical to clinical models.

Several pilot trials have revealed the promising potential of noninvasive VNS for depression. Hein and colleagues were the first to investigate the antidepressant effects of taVNS for major depression in a double-blind RCT [63]. Thirty-seven depressed patients were randomized to active or sham taVNS for 2 weeks. After 2 weeks, the active taVNS group showed significantly better outcomes (as measured by the Beck Depression Inventory [BDI]) than the control group. There was no significant difference between the two groups on a different depression rating scale (Hamilton Depression Rating Scale [HAM-D]).

In 2016, Rong completed a non-randomized controlled pilot trial investigating taVNS for MDD [64]. The active group in this study received 12 weeks of stimulation and the control group received 4 weeks of sham followed by 8 weeks of taVNS. The participants in this study completed all of the stimulation sessions at home, and taVNS was self-administered. After the 4-week sham-controlled period, the active taVNS group had significant improvements on the HAM-D compared to the sham group. Clinical improvements continued up until week 12 of the study.

Similarly, Trevizol and colleagues reported on an open-label trial of taVNS for MDD where patients received 10 treatments over the course of 2 weeks [65]. There was a significant decrease in HAM-D scores overall (mean 27.9 at baseline, 8.2 at day 10), and this reduction was sustained at a 45-day follow-up (8.75). More recently, a randomized trial compared taVNS to citalopram in the treatment of MDD [66]. Treatment with taVNS demonstrated similar improvements in depressive symptoms compared to citalopram. Researchers are beginning to study the effects of taVNS on depression in pediatric populations [67] and secondary causes of depression, like post-stroke depression [68].

The early studies discussed in this section have shown promise with modest effect sizes. While the use of transcutaneous VNS is still quite young, excitement about the future of this modality is growing. Investigations in this area will be important to determine whether noninvasive VNS itself can serve as a treatment for depression, or whether taVNS response may be a good predictor of response in patients who will go on to receive iVNS.

## VNS for obesity

### Implanted VNS

Over the past 50 years, the number of people who are overweight or obese has risen dramatically in most parts of the world [69]. Obesity often carries with it other comorbidities and increases the risk of other metabolic and cardiovascular diseases. The increased mortality and overall cost burden to global healthcare systems brings about the need for solid and viable treatments for obesity. iVNS has been studied as a potential therapeutic option for patients suffering from obesity, and the evidence is discussed in this section.

Several methods have been used to investigate of iVNS for obesity. Along with implanted cervical and noninvasive cutaneous, another form of iVNS, known as infradiaphragmatic iVNS or vagal nerve blocking, has also been explored as a potential treatment option. Vagal nerve blocking requires laparoscopic surgery in which electrodes are placed around the anterior and posterior vagus nerves at the gastroesophageal junction [70]. These electrodes are connected to a pulse generator that is implanted subcutaneously on the thoracic side wall.

In a study originally designed to elucidate the efficacy of invasive cervical iVNS for depression (parameters similar to other depression studies: duty cycle of 30 s on, 5 min off; 250–500 µs pulse width; 30 Hz frequency; 0.25–1.5 mA current amplitude), depressed patients lost an average of 7 kg, with 2–3-point reductions in body mass index (BMI) [71].

The Maestro System is a vagal nerve blocking device that has been FDA-approved for the treatment of obesity. The system uses a duty cycle of 5 min on, 5 min off, and treats within a range of 1–6 mA at a frequency of 5000 Hz [53]. Four case series were published between 2008 and 2017, all with positive findings denoted by percent excess weight loss (%EWL). In these case series, the %EWL ranged between 14.2% and 22% [72, 73, 74, 75]. The success in the early case series led to the completion of three RCTs from 2012 to 2016 [70, 76, 77]. Most of these studies included a behavioral intervention, such as weight management education or counseling, adjunctive to active or sham iVNS treatment.

The first RCT, led by Sarr and colleagues, found no significant difference in %EWL between active and sham stimulation, but demonstrated a significant reduction in EWL over time [76]. The other two RCTs found significant differences in mean %EWL between active and sham conditions, with %EWL ranging from 24.4% to 33% in the active groups and 15.9% to 19% in the sham groups. In one study in particular, response rates were as high as 59% in the active group and 41% in the sham groups [77]. It is important to note the side effect profile of the abdominal vagus system, which is associated with higher rates of gastrointestinal side effects (heartburn, nausea, dysphagia, and abdominal pain) than other methods of iVNS. This is likely due to the location of implantation and stimulation. Based on the evidence discussed in this section, iVNS was approved for the treatment of morbid obesity by the FDA in 2015.

### Noninvasive VNS

A separate study explored the potential of noninvasive VNS as a treatment for glucose intolerance, collecting measures of BMI as secondary outcomes [78]. The study found no significant differences in BMI between active and sham stimulation. However, they found that 12 weeks of postprandial taVNS significantly reduced 2-h glucose tolerance and systolic blood pressure. While newer, noninvasive VNS modalities offer hope that clinicians will one day be able to offer these simpler, safer options to patients, additional data are needed to understand whether they are as clinically effective in the treatment of obesity as their invasive counterparts.

## Motor rehabilitation

VNS for post-stroke motor rehabilitation is a brilliant example of translating VNS paradigms from animal models to humans. Stroke is the second leading cause of death and disability worldwide, and prevalence is expected to grow by 2030 [79]. Motor rehabilitation is effective in helping stroke patients regain function and improving quality of life. However, many patients live with deficits beyond the rehabilitation period. Novel methods to enhance the effects of physical rehabilitation in the post-stroke period are essential in reducing this burden on stroke survivors, caregivers, and society. Based on promising preclinical evidence, invasive and noninvasive forms of VNS have now been studied as potential treatments for post-stroke recovery in humans.

### Implanted VNS

Invasive cervical iVNS trials evaluated the impact of iVNS paired with physical rehabilitation versus physical rehabilitation with sham stimulation (Table 3). Stimulation parameters were consistent across studies; over 6 weeks, patients received 18 sessions, with the following parameters: 0.8 mA current amplitude, 0.1 ms pulse width, 30 Hz frequency, and 0.5 s duration [59, 60]. Stimulation was delivered paired with repetitive movements. Data from small pilot and large multicenter RCTs produced similar trends: paired iVNS and motor rehabilitation compared to rehabilitation with sham iVNS was associated with clinically meaningful improvements in participants with moderate to severe arm impairment after ischemic stroke [80]. In one study, the active iVNS group had two to three times greater improvements than the sham iVNS group, in a population that was at least 9 months post-stroke [3]. In a population with extremely limited therapeutic options, these results may help fill the void. Overall, the results of these studies are quite exciting, with more than ample evidence of iVNS and its ability to enhance post-stroke recovery. Next steps in this area include assessing whether the model can be used earlier in the post-stroke recovery course and whether these results are applicable in other populations (other types of strokes, other neurological disorders). In 2021, the MicroTransponder Vivistim Paired VNS system (Vivistim system) was approved by the FDA for the treatment of moderate to severe upper extremity motor deficits associated with chronic ischemic stroke.

**Table 3:** VNS for post-stroke motor rehabilitation trials

Authors	Study/sample size	Parameters (active)	Implanted/transcutaneous	Primary outcome	Conclusions
Dawson et al.	21 participants with ischemic stroke more than 6 months earlier and moderate to severe upper limb impairment	PW: 100 μs Freq: 30 Hz On/off: 500 ms during movement I: 0.8 mA	Implanted	Change in Fugl-Meyer Assessment–Upper Extremity (FMA-UE) scores in VNS plus rehabilitation group vs. rehabilitation alone	In the intention-to-treat analysis, there was no change in the FMA-UE score between groups
Dawson et al.	RCT of 108 participants with moderate to severe arm weakness, at least 9 months after ischemic stroke	PW: 100 μs Freq: 30 Hz On/off: 0.5 s during movement I: 0.8 mA	Implanted	Change in impairment measured by the Fugl-Meyer Assessment–Upper Extremity (FMA-UE) score	The mean FMA-UE score increased significantly more in the VNS group than in controls; a clinically meaningful response on the FMA-UE score was achieved in 47% with VNS vs. 24% in controls
Capone et al.	14 patients with either ischemic or hemorrhagic chronic stroke	PW: 0.3 ms Freq: 20 Hz On/off: 30 s train/5 min I: varied	Transcutaneous	Change in upper extremity Fugl-Meyer score between groups receiving robot-assisted therapy with real vs. sham VNS	Fugl-Meyer scores were significantly better in the real group than the sham group after 10 days of treatment
Redgrave et al.	13 participants at more than 3 months post-ischemic stroke with residual upper limb dysfunction	PW: 0.1 ms Freq: 25 Hz On/off: during movement I: Varied	Transcutaneous	Change in Fugl-Meyer Assessment–Upper Extremity scores after receiving UE rehab + tVNS	There was a significant change in the FMA-UE score, with a mean increase per participant of 17.1 points
Baig et al.	12 participants at more than 3 months post-ischemic stroke with residual upper limb weakness	PW: 0.1 ms Freq: 25 Hz On/off: during movement I: varied	Transcutaneous	Change in upper limb Fugl-Meyer score from baseline following motor rehab + tVNS for 6 weeks	64% of participants regained some sensation post-intervention, with maximal increase in FMA-UE sensation score seen in the patient with the greatest improvement in motor function
Wu et al.	21 subacute ischemia stroke patients with single upper limb motor function impairment	PW: 0.3 ms Freq: 20 Hz On/off: 30 s/5 min I: varied	Transcutaneous	Change in upper extremity Fugl-Meyer Assessment, the Wolf motor function test (WMFT), the Functional Independence Measure (FIM), and Brunnstrom stage from baseline in rehab + active tVNS vs. rehab + sham group after 15 days of treatment	The FMA-UE, WMFT, and FIM scores were significantly higher than before treatment, and there was a significantly greater improvement of those measurements in the tVNS group compared with sham-tVNS group
Badran et al.	16 post-stroke patients undergoing rehab	PW: 500 μs Freq: 25 Hz On/off: 5 s increments during movement I: 1-3 mA	Transcutaneous	Change in upper limb Fugl-Meyer Assessment, Wolf motor function test	Improved upper limb Fugl-Meyer scores compared to sham

*PW* pulse width

### Noninvasive VNS

Noninvasive VNS has also been studied as a potential therapeutic tool for post-stroke motor recovery. The data in this area are limited but show early promise (see Table 3). All the noninvasive studies have utilized taVNS, but their parameters have differed slightly [81, 82, 83, 84]. Three out of four transcutaneous studies targeted the left cymba concha, while one out of the four targeted the left acoustic meatus. One open-label trial saw 87% of participants (*n* = 13) achieve a clinically relevant increase in mobility as measured by the Fugl Meyer Assessment–Upper Extremity (FMA-UE) [82]. Three small RCTs have demonstrated similar results, with significant improvements seen in stroke patients receiving active taVNS with physical rehabilitation compared to those receiving sham stimulation with physical rehabilitation [81, 83, 84]. Overall, these studies confirmed the exciting results seen in the implanted cervical VNS population, with active stimulation producing 2–3 times greater improvements than physical rehabilitation alone. Larger trials at multiple centers will be needed before noninvasive options are approved for treatment.

A recent pilot study of closed-loop taVNS (called motor-activated auricular vagus nerve stimulation, or MAAVNS) is moving this area of study towards more personalized treatment [13]. MAAVNS is a closed-loop system designed to improve upper limb function by delivering stimulation precisely in response to movement detected by surface electromyography (EMG) sensors. MAAVNS is individualized to each patient, and the pilot trial demonstrated promising results. The pilot investigated the effects of paired (MAAVNS) and unpaired taVNS on upper extremity motor recovery scores, finding that both groups improved, with a greater effect size in the MAAVNS group.

Another trial by the same group investigated the effects of unilateral versus bilateral taVNS on neural activity in chronic stroke patients [85]. The group measured blood-oxygenation-level-dependent (BOLD) signal propagation in response to ipsilesional versus contralesional versus bilateral versus sham stimulation. The findings suggest that ipsilesional taVNS may be optimal for treatment in post-stroke recovery, as ipsilesional taVNS produced the greatest brain activation in key areas involved in stroke recovery as well as producing greater task-related activation.

## Headache

Another area that has generated significant interest in VNS research and clinical applications is in the treatment of headaches. Investigations in this domain have mainly used noninvasive VNS modalities (transcutaneous auricular and cervical VNS). One device (marketed as gammaCore) has been cleared by the FDA for acute and preventive treatment of cluster headache and acute treatment of migraine in adults. The gammaCore device uses a form of transcutaneous cervical VNS (tcVNS) that delivers electrical stimulation consisting of five 5000 Hz pulses repeated at a rate of 25 Hz [66]. This section will broadly overview VNS research into the treatment of headaches and briefly summarize future directions in which the field is heading.

### Implanted VNS

Reductions in headache severity and frequency were reported in preliminary case series using invasive iVNS for the treatment of depression or epilepsy [86]. However, prospective investigations of vagal stimulation with headaches as the primary diagnosis have only used noninvasive VNS methods.

### Noninvasive VNS

The advent of noninvasive VNS has moved this area forward, with many positive RCTs to date. tcVNS, under investigation as gammaCore, has been the most thoroughly studied as an acute treatment for migraine and cluster headache, as well as prophylactic treatment. Early case series of tcVNS for acute migraine showed a significant reduction in duration of pain intensity and headache remission rates [87]. In one open-label pilot study, 22% of participants were found to be pain-free rate at 2 h, comparable to abortive medications commonly used for migraine (i.e. triptans) [88]. In a follow-up RCT known as the PRESTO study, the effects of tcVNS on acute migraine were evaluated in a group of 248 patients [89]. Active stimulation consisted of the parameters described above, delivered for a duration of 120 s, while the sham group received 0.1 Hz biphasic stimulation. The data from this study also showed tcVNS to have equivalent efficacy as medication for acute migraine [89]. Interestingly, tcVNS was superior to sham at time points 30 and 60 min, but not at 120 min, although the pain-free responder rate was similar to what is seen with triptans and oral nonsteroidal anti-inflammatory drugs (NSAIDs).

Studies of tcVNS for cluster headache have also yielded positive results. An open-label pilot found positive results for acute cluster headaches (47% of attacks stopped in 11 min or less) and preventive treatment (reduction in mean attack frequency) [88]. The open-label trial was followed by two RCTs (ACT1 and ACT2) [90, 91]. The ACT1 trial found that active treatment significantly increased the response rate (defined as the proportion of subjects who achieved pain relief) compared to sham stimulation in the episodic cluster headache group, but not the chronic group [90]. The ACT2 study had similar findings, with benefits seen only in the episodic group and not in those with chronic cluster headache. In contrast, the PREVA study in 2016 found a significant difference between active and sham stimulation in the prevention of chronic cluster headache attacks [91].

tcVNS has also been studied as a preventive treatment for migraine, but with mixed results. The PREMIUM trial is the largest RCT to date [92]. Diener’s study consisted of a 12-week double-blind phase followed by a 24-week open-label phase; throughout both phases, a total of 332 patients self-administered stimulation for 120 s twice daily (6–8 h apart). The final analysis, however, did not demonstrate a statistically significant difference between the active and sham groups regarding migraine frequency or reduction.

While other forms of noninvasive VNS, like taVNS, have been investigated for different types of headaches, less evidence exists for these modalities. Straube and colleagues led an investigation of 1 Hz taVNS for the prevention of chronic migraine and found that active stimulation was associated with a reduction in number of headache days compared to sham stimulation [93]. In a separate study, 1 Hz taVNS significantly reduced migraine days, pain intensity, and migraine attack time, while also implicating direct thalamocortical modulation, as measured by change in BOLD functional magnetic resonance imaging (fMRI) signaling, as potentially responsible for the positive therapeutic effects of active taVNS on migraine [94].

## Exploratory pilot trials

VNS is being studied as a potential therapeutic for a variety of other conditions, including pain, inflammatory disorders, cardiovascular diseases, and gastrointestinal disorders. These conditions have been predominately studied using noninvasive VNS. This section will briefly overview exploratory work that has been conducted in each category.

### Pain disorders

The analgesic effects of VNS have been described since the modality’s inception. The interplay between various VNS parameters and their behavioral effects remains to be determined. Here, we give a brief overview of VNS for pain studies. VNS for headache and migraine studies is discussed in the headache section above.

VNS was found to be effective for a variety of conditions associated with chronic pain. In addition to the headache and migraine studies, the antinociceptive effects of VNS have been demonstrated for conditions such as fibromyalgia, pancreatitis, irritable bowel syndrome (IBS), esophageal pain, and polymyalgia rheumatica [7]. The advent of noninvasive VNS has allowed more feasible and less costly study of VNS and its effects on these pain-related disorders. The Cerbomed Nemos taVNS device (tVNS Technologies, Erlangen, Germany) received European certification for chronic pain in 2012. However, several iVNS trials have demonstrated analgesic effects. For example, an interesting trial of iVNS (for coronary artery disease [CAD]) abolished angina at rest, as well as reducing heart rate and blood pressure [95].

taVNS successfully decreased pain and fatigue in a group of patients (*N* = 18, randomized 2:1 to receive active) with systemic lupus erythematosus (SLE) [96]. Bellocchi found similar results for SLE-related pain, using taVNS as an adjunct to usual treatment [97]. The group also found downregulated interleukin-6 levels in the active group compared to sham. Interestingly, there was no significant effect on quality-of-life scales or heart rate variability, which have been seen in other VNS studies (e.g., for major depression and epilepsy).

taVNS has also been shown to relieve abdominal pain and constipation associated with IBS [98]. Kovacic et al. found that impaired cardiac vagal regulation (measured by vagal efficiency) predicted pain improvement associated with taVNS [99]. tcVNS was investigated as a potential therapeutic for pain associated with chronic pancreatitis, but no difference was found in pain scores between active and sham treatment [100]. In a single-blind sham-controlled study, taVNS combined with deep slow breathing enhanced gastroduodenal motility and antral contractions but did not demonstrate any significant effect on pain thresholds [101]. Napadow and colleagues described a modified form of taVNS called respiratory-gated auricular vagal afferent nerve stimulation (RAVANS), which they used to study chronic pelvic pain due to endometriosis. They demonstrated reductions in evoked pain intensity and temporal summation of mechanical pain, as well as anxiety [102].

Vagal modulation of pain has also been studied in healthy subjects. Reports indicate that taVNS may increase mechanical and pressure pain thresholds as well as mechanical pain sensitivity [103]. Alt and colleagues demonstrated reductions in pain unpleasantness ratings due to taVNS [104]. taVNS has also been shown to decrease acid-induced esophageal hypersensitivity [105]. However, some trials did not show any effect of taVNS on pain in healthy adults [31].

### Inflammatory disorders

VNS modulation of the neuroimmune system has been well studied in animal models, but less so in humans. VNS has been shown to decrease inflammation associated with irritable bowel disease, as well as inflammation associated with stroke, traumatic brain injury (TBI), and depression [106]. Most studies to date in this arena have been preclinical animal models, but noninvasive VNS represents an opportunity for further translation of these models into the clinical domain.

In a study of iVNS for Crohn’s disease, five out of nine patients experienced remission of symptoms, as well as decreased levels of inflammatory markers (C-reactive protein and calprotectin) [107]. Another pilot trial had similar, although less impressive, results. Four out of 16 patients implanted with iVNS experienced remission of Crohn’s symptoms [108]. Additionally, iVNS decreases cytokine production and attenuates disease severity in rheumatoid arthritis. Promising results have been seen in the use of taVNS in small cohorts of patients with various inflammatory disorders. In a study of patients with systemic lupus erythematosus (SLE), plasma levels of Substance P were significantly reduced after 5 days of taVNS [96]. taVNS reduced pain scores, as well as downregulating IL-6, in patients with systemic sclerosis [97]. Additionally, patients with sepsis showed a reduction in inflammatory cytokines after five consecutive days of taVNS [109]. taVNS decreased levels of inflammatory cytokines associated with myocardial ischemia and reperfusion, atrial arrhythmias, and heart failure [110]. An interesting pilot trial of taVNS for symptoms associated with long COVID demonstrated decreases in anxiety and fatigue, which was hypothesized to be due to a decrease in inflammation associated with the development of long COVID [111].

### Cardiovascular disorders

Several large trials have investigated the effectiveness of invasive VNS for heart failure [112, 113, 114]. Unfortunately, these trials did not report significant differences in outcomes between active and sham groups. Premchand and colleagues demonstrated improved left ventricular ejection fraction by 4.5% in patients 10 weeks after the implantation of iVNS in a study of 60 patients with heart failure [115]. Zamotrinsky and colleagues found that transcutaneous VNS treatment abolished angina at rest and reduced heart rate and blood pressure [116]. They also found improvements in left ventricular ejection fraction. Yu found similar results—taVNS reduced ventricular arrhythmia and improved left ventricular ejection fraction [117].

Additionally, VNS has been shown to work through other cardiovascular mechanisms, including lowering sympathetic activity and improving baroreflex control. [32, 33]. These could be potential mechanisms for the treatment of conditions characterized by sympathetic overactivity, like hypertension and chronic kidney disease. Additionally, cardiovascular mechanisms, including lowering of sympathetic activity, may play a role in the psychotherapeutic effects of VNS.

### Gastrointestinal disorders

VNS has been studied in a variety of gastrointestinal disorders. Implanted VNS is FDA-approved for obesity but has early evidence for other gastrointestinal disease. It is likely effective for gastrointestinal disease through a combination of its parasympathetic (autonomic afferent and efferent) functions and its ability to modulate the neuroimmune and neuroendocrine systems. All the early studies for gastrointestinal disease have used noninvasive VNS. After 4 weeks of taVNS, patients with constipation-predominant irritable bowel syndrome (IBS-C) had decreased abdominal pain and increased frequency of complete bowel movements [98]. Patients with Parkinson’s disease who received taVNS had improved scores on the Gastrointestinal Symptom Rating Scale [118]. Zhang and colleagues investigated the effectiveness of a similar treatment, transcutaneous electrical acustimulation (TEA), on gastric motility and gastroesophageal reflux disease [119]. They found that TEA had positive effects on reflux-related symptoms and motility. In a trial using tcVNS (gammaCore), 10 out of 23 patients with drug-resistant gastroparesis saw a response (measured as improvements in nausea/vomiting, postprandial fullness/early satiety, and bloating) [120]. Another pilot trial had similar results, with 40% of patients reporting improvement in symptoms related to gastroparesis, including accelerated gastric emptying [121].

## Future directions

As reviewed in the prior sections, VNS is emerging as a promising modality with the ability to treat a wide variety of medical conditions and neuropsychiatric disorders. The field is still young and there are large gaps in the literature. We have highlighted these gaps in each of the preceding sections, but here we give readers a summary and overview of these areas for future improvements.

As highlighted in the epilepsy and motor rehabilitation sections, closed-loop VNS systems are beginning to be studied and have made their way into the clinical domain [12, 13]. The success of responsive VNS, which sends out trains of stimulation in response to ictal tachycardia, in further decreasing seizure burden compared to standard VNS showcases the promise of closed-loop systems [12]. Creating personalized VNS paradigms will require greater understanding of the disorders we are treating. VNS combined with other techniques, such as brain imaging or electroencephalography (EEG), will aid in developing personalized treatments. Additionally, noninvasive forms of VNS will be useful in testing, optimizing, and scaling up these novel paradigms.

Noninvasive forms of VNS, like taVNS and tcVNS, have created opportunities to more deeply understand how VNS works [10, 11, 30]. Noninvasive VNS has allowed researchers to study the physiologic effects of vagal stimulation in a feasible and cost-friendly manner. Head-to-head trials of noninvasive VNS and iVNS will enable us to understand how these modalities differ. Noninvasive VNS may be useful in predicting response to VNS and prognosis. Additionally, noninvasive VNS will be useful for optimizing parameters. The wearable aspect of noninvasive VNS will allow more clinicians and patients to access this technology and has opened the door for studying VNS in novel settings, including at-home treatment [122].

Finally, VNS has momentum geared towards future approvals. As highlighted in the previous section, VNS has accumulating evidence in many different medical conditions, including pain disorders, inflammatory disorders, cardiovascular disorders, and gastrointestinal disorders. As we understand more about how VNS works, we will also learn more about the pathophysiology of the disorders we are trying to treat.

## Conclusion

Inevitably, more data will help shape our understanding of how these novel treatments can be made more effective. As in other areas of VNS research, future areas for improvement are in our understanding of how parameters interact with disease state. VNS has an infinite number of parameter combinations, as clinicians can adjust frequency, pulse width, duty cycle, and duration of stimulation. Understanding how these parameter combinations affect treatment outcomes is of utmost importance. Additionally, noninvasive VNS has opened the door for further study of the effects of VNS. This includes translation of the wealth of evidence in animal models to human clinical research, as well as further study of how VNS may be an effective treatment for other medical and neuropsychiatric disorders. Discovery of reliable biomarkers will be crucial in aiding the development of personalized VNS treatments, a revolution that is already occurring in other areas of brain stimulation.

In conclusion, the history of VNS spans four decades but has already accomplished a great deal. To date, VNS has gained approvals from the FDA for medication-refractory epilepsy, treatment-resistant depression, obesity, post-stroke motor rehabilitation, and migraine and cluster headache. The development of novel noninvasive forms of VNS promises to further advance the field’s evolution.
